# Linc00659, a long noncoding RNA, acts as novel oncogene in regulating cancer cell growth in colorectal cancer

**DOI:** 10.1186/s12943-018-0821-1

**Published:** 2018-03-10

**Authors:** Kuo-Wang Tsai, Yi-Hao Lo, Hsuan Liu, Chung-Yu Yeh, You-Zuo Chen, Chao-Wen Hsu, Wei-Shone Chen, Jui-Ho Wang

**Affiliations:** 1 Department of Medical Education and Research, Kaohsiung Veterans General Hospital, Kaohsiung, 813 Taiwan, Republic of China; 2 Institute of Biomedical Sciences, National Sun Yat-Sen University, Kaohsiung, Taiwan; 3 Department of Chemical Biology, National Pingtung University of Education, Pingtung, Taiwan; 4 Department of Family Medicine, Zuoying Branch of Kaohsiung Armed Forces General Hospital, Kaohsiung, Taiwan; 5 Department of Biochemistry, College of Medicine, Chang Gung University, Kueishan, Taoyuan, Taiwan; 6 Division of Colon and Rectal Surgery, Department of Surgery, Chang Gung Memorial Hospital, Linkou, Taiwan; 7 Division of Colorectal Surgery, Department of Surgery, Kaohsiung Veterans General Hospital, Kaohsiung, 813 Taiwan, Republic of China; 8 School of Medicine, National Yang-Ming University, Taipei, Taiwan; 9 Department of Surgery, Veterans General Hospital, Taipei, Taiwan, Republic of China

**Keywords:** Colorectal carcinoma, lncRNA, Noncoding RNA

## Abstract

**Background:**

Colorectal cancer (CRC) is one of the most common cancers and causes of cancer-related death worldwide. In patients with CRC, metastasis is a crucial problem that leads to treatment failure and is the primary cause of the lethality of colon cancer. Long noncoding RNAs (lncRNAs) have recently emerged as critical molecules in the development, cell growth, apoptosis, and metastasis of CRC.

**Method:**

We investigated the transcriptome profiles of human lncRNAs in the primary tumor tissues and in the corresponding normal mucosa of two patients with CRC by using a microarray approach. The expression levels of lncRNAs were verified in colon cancer by real-time PCR. Using bioinformatics approach to illustrate putative biological function of Linc00659 in colon cancer. The effects of Linc00659 on cell growth, proliferation, cell cycle and apoptosis were studies by in vitro assays.

**Results:**

Our data revealed that compared with adjacent normal tissues, 201 lncRNAs were deregulated (fold change ≥ 4 or ≤ 0.25) in CRC tissues. Among them, the expression levels of Linc00659 were significantly increased in colon cancer, and high expression levels were correlated with poor survival in patients with CRC. Bioinformatics analysis results indicated that Linc00659 was significantly coexpressed with cycle-related genes in CRC. Linc00659 expression knockdown could significantly suppress colon cancer cell growth by impairing cell cycle progression. In addition, our results showed that Linc00659 expression knockdown could accelerate cell apoptosis in colon cancer cells treated with chemotherapy drugs. Meanwhile, our results also demonstrated that silencing of Linc00659 expression leads to cell growth inhibition and induced apoptosis, possibly by suppressing PI3K-AKT signaling in colon cancer.

**Conclusion:**

Linc00659 is a novel oncogenic lncRNA involved in colon cancer cell growth by modulating the cell cycle. Our findings give an insight into lncRNA regulation and provide an application for colon cancer therapy.

**Electronic supplementary material:**

The online version of this article (10.1186/s12943-018-0821-1) contains supplementary material, which is available to authorized users.

## Background

Colorectal cancer (CRC) is one of the most common cancers and causes of cancer-related death worldwide [1, 2]. The major causes of the lethality of colon cancer are distant metastasis and drug resistance, which usually lead to a poor survival rate [3]. In patients diagnosed as having advanced-stage CRC, distant metastasis is frequently observed, leading to poor therapeutic effects of chemotherapy, radiotherapy, and surgery [4]. Therefore, developing a diagnostic biomarker for early detection of CRC or as a therapeutic target will improve the survival rate of patients with CRC.

High-throughput genome-scale approaches have revealed that a large amount of transcripts are produced from more than 80% of human genomic DNA, and only 1–2% of the human genome is composed of protein-coding genes [5]. Therefore, the human genome comprehensively harbors numerous transcripts with no protein-coding function. Recent studies have shown that numerous noncoding RNAs (ncRNAs) play a critical role in regulating various cellular processes [6]. Long noncoding RNAs (lncRNAs) are a group of long RNA transcripts that are more than 200 nucleotides in length; however, they lack protein translation ability. An increasing body of evidence indicates that lncRNAs play a critical role in regulating various cellular processes, including cell development and growth, the cell cycle, and cancer metastasis, by (1) regulating protein-coding gene expression, (2) modifying epigenetic regulation, (3) modulating the alternative splicing process, and (4) acting as decoys that titrate microRNAs [7, 8]. To date, some studies have reported that numerous lncRNAs, including HOTAIR, PCAT-1, PVT-1, H19, BANCR, 91H, CCAT1, and MALAT1, were dysregulated in colon cancer [9–21]. These dysfunctional lncRNAs have been reported to have tumor-suppressive or oncogenic roles in regulating cell growth, the cell cycle, and cell migration in CRC. However, the function of most lncRNAs remains largely unknown in CRC. In this study, our objective was to identify dysregulated lncRNAs in CRC. For this purpose, we compared the transcriptome profiles of primary tumor with adjacent normal tissue from two patients by using a microarray approach. We further evaluated the expression levels and biological role of Linc00659 in CRC through bioinformatics and an experimental approach. Our finding revealed that Linc00659 is a novel oncogenic lncRNA that regulates CRC cell growth by impairing cell cycle progression, and thus may be a potential therapeutic target for gene therapy.

## Methods

### Cell line

Eight human CRC cell lines, namely DLD-1, colo205, colo320DM, LS174T, HCT116, SW620, LOVO, and HT29, were obtained from the American Type Culture Collection. Cells were cultured in high-glucose Dulbecco’s modified Eagle’s medium (Invitrogen, Grand Island, NY, USA) with 10% fetal bovine serum (Hyclone Laboratories, Inc., South Logan, UT, USA) and 1% penicillin/streptomycin (Gibco, Thermo Fisher Scientific Inc., Waltham, MA, USA) in plastic tissue culture plates in a humidified atmosphere containing 5% CO_2_ at 37 °C.

### Clinical samples

Colon cancer tissue and the corresponding adjacent normal mucosa samples were obtained from 91 patients with CRC who underwent surgery at the Department of Surgery, Taipei Veterans General Hospital, Taiwan. Informed consent was obtained from all patients.

### Extraction of RNA

Total RNA from tissue was extracted using TRIzol reagent (Invitrogen, Grand Island, NY, USA), according to instructions in the user’s manual. In brief, tissue samples were homogenized in 1 mL TRIzol reagent and mixed with 0.2 mL chloroform to extract protein, before RNA was precipitated using 0.5 mL isopropanol. The concentration, purity, and amount of total RNA were determined using a Nanodrop 1000 spectrophotometer (Nanodrop Technologies Inc., Wilmington, DE, USA).

### LncRNA profiling and pathway enrichment analysis

Total RNAs of four clinical samples were extracted, involving two primary CRC tissues and two corresponding adjacent normal tissues obtained from two patients with CRC, respectively. The microarray experiments and data analysis were performed by Welgene Biotech (Taipei, Taiwan) using the Agilent Oligo Chip (Agilent SurePrint G3 Human V2 GE; including protein-coding genes and lncRNAs). Differential expression protein-coding genes (tumor versus normal ≥ 2 and ≤ 0.5) were selected from microarray data and candidate genes were mapped onto the Kyoto Encyclopedia of Genes and Genomes pathways based on the Enzyme Commission numbers by using the R package SubpathwayMiner v.3.1. Subsequently, a hypergeometric test was performed to identify significantly enriched pathways and calculate the false-positive discovery rate in the false discovery rate-corrected *q* value.

### Expression data from the cancer genome atlas

The transcriptome expression profiles of colon cancer were downloaded from The Cancer Genome Atlas (TCGA) data portal (https://cancergenome.nih.gov). The expression profiles of 616 colon cancer tissues and 51 adjacent normal tissues were obtained from TCGA data portal. In this study, the transcriptome profiles of 29 N-T pairs were used for coexpression analysis and 616 cases were included in the survival analysis.

### Reverse transcription and real-time polymerase chain reaction

In this reaction, 2 μg of total RNA was reverse transcribed with random primers (Invitrogen; Thermo Fisher Scientific Inc., Waltham, MA, USA) and SuperScript IV Reverse Transcriptase according to the user’s manual (Invitrogen; Thermo Fisher Scientific Inc., Waltham, MA, USA). The reaction was performed with incubation at 42 °C for 1 h, and the enzyme was subsequently inactivated by incubation at 85 °C for 5 min. cDNA was used for real-time polymerase chain reaction (PCR) analysis with gene-specific primers, and gene expression was detected using a Fast SYBR Green Master Mix (Applied Biosystems; Thermo Fisher Scientific Inc., Waltham, MA, USA). The expression of lncRNA was normalized to that of glyceraldehyde 3-phosphate dehydrogenase (GAPDH; △*Ct* = target lncRNA *Ct* – GAPDH *Ct*). The individual primers used in this study were as follows:

- Linc00659-F: 5′-ACCCCTGAAGGACCATATCCA-3′;
- Linc00659-R: 5′-GGCTCGGCTGTGTCTCAAG-3′;
- MNX1-AS1-F: 5′-GCTCTGCAGGTCGAACCTTA-3′;
- MNX1-AS1-R: 5′-CCCGCAGGCTAGTGTCTATC-3′;
- Loc339524-F: 5′-TGGCAGCAAGTCCATCTCAT-3′;
- Loc339524-R: 5′-CTTTGACACGGCTGACTTGG-3′;
- Linc00675-F: 5′-GTTGCAGCTTCCACCTAGCA-3′;
- Linc00675-R: 5′-TCTCGGACATCCTCGTGAGT-3′;
- GAPDH-F: 5′-TGCACCACCAACTGCTTAGC-3′;
- GAPDH-R: 5′-GGCATGGACTGTGGTCATGAG-3′.

### Small interfering RNA transfection

Small interfering RNA (siRNA) oligonucleotides targeting Linc00659 (si-Linc00659, sense: 3′-TTGGGAGGAACACGAAGUCUU-5′ and antisense: 5′-UUCUGAAGCACAAGGAGGGTT-3′) and a scrambled oligo as a negative control were designed and synthesized by GenDiscovery Biotechnology (Taipei, Taiwan). Cells were transfected with a final concentration (10 mM) of individual siRNA or control using Lipofectamine RNAiMAX (Invitrogen; Thermo Fisher Scientific Inc., Waltham, MA, USA). After transfection for 24 h, the RNA was extracted and knockdown efficiency was evaluated by real-time PCR.

### Stable Linc00659 knockdown with short hairpin RNA

Stable HCT116 cells with Linc00659 knockdown were generated by infecting colon cancer HCT116 cells with lentiviruses expressing sh-Linc00659 in the presence of 8 μg/mL polybrene for 24 h followed by puromycin (4 μg/mL) selection for 3–5 days. The shLuc vector targeting the luciferase gene provided puromycin resistance and was used as the control. Linc00659 expression was confirmed by a real-time PCR assay. In this study, we designed two shRNA sequence targeted Linc00659, and individual sequence of shRNA used for constructs in this study were as follows:

- Sh-Linc00659#1:
  - Sense:5’-CCGGTCCCTCCTTGTGCTTCAGAAGCATTCAAGAGATGCTTCTGAAGCACAAGGAGGGTTTTTTG-3′.
  - Antisense:5’-AATTCAAAAAACCCTCCTTGTGCTTCAGAAGCATCTCTTGAATGCTTACGAAGCACAAGGAGGGA-3’.
- Sh-Linc00659#2:
  - Sense:5’-CCGGTCCGGGGTAGAGAGCAGTGAGGAACTCGAGTTCCTCACTGCTCTCTACCTTTTTGG-3′.
    Antisense:5’-AATTCCAAAAAGGTAGAGAGCAGTGAGGAACTCGAGTTCCTCACTGCTCTCTACCCCGGA-3’.

### Proliferation

For cell proliferation analysis, 2500 living cells were transfected with either siLinc00659 or a scrambled control and plated onto 96-well plates. Cell growth was determined at 0, 1, 2, 3, and 4 days. Cell viability was determined using a CellTiter-Glo One Solution cell proliferation assay (Promega Corporation, Madison, WI, USA), which was performed according to the manufacturer’s instructions.

### Soft agar assay

In brief, this assay was performed as follows: The base agar layer was prepared with 1% agar (Laboratorios CONDA, Torrejón de Ardoz, Madrid, Spain) dissolved in 1.5 mL phosphate-buffered saline on 6-well plates; subsequently, 1.5 mL of 0.7% agarose (Invitrogen, Grand Island, NY, USA) solution containing the cells (10,000 cells per well) was inoculated on top of the base agar layer. After allowing the solution to harden, 0.5 mL of fresh medium was added to the top of the hard agar layer. After 3–4 weeks, agar plates were stained with iodonitrotetrazolium chloride (Santa Cruz Biotechnology, Santa Cruz, CA, USA) and colonies were counted.

### Colony formation assay

A total of 4000 cells were seeded on each well of a 6-well plate, and the cells were transfected with individual siRNA oligonucleotides by using Lipofectamine RNAiMAX (Invitrogen; Thermo Fisher Scientific Inc., Waltham, MA, USA) in the 6-well plate. After incubation at 37 °C for 3 days, the cultured medium was replaced with a new medium. The cells were allowed to incubate at 37 °C for 10 days. Cell culture plates containing colonies were fixed with 4% formaldehyde for 2 min and colonies were stained with crystal violet solution (containing 0.5% crystal violet, 5% formaldehyde, 50% ethanol, and 0.85% sodium chloride) for 2 h. Wells were rinsed with H_2_O after air drying. The crystal violet staining of cells from each well was solubilized using 1 mL per well of 10% acetic acid, and the absorbance (optical density) of the solution was measured on a spectrophotometer at a wavelength of 595 nm.

### Cell cycle analysis

A total of 1 × 10^6^ cells were collected and mixed with 70% ethanol in a fixative at − 20 °C overnight. Cells were then stained with 4′,6-diamidino-2-phenylindole (ChemoMetec, Gydevang, Lillerød, Denmark) and detected using NucleoView NC-3000 software (ChemoMetec, Gydevang, Lillerød, Denmark).

### Apoptotic cells stained with Annexin V, propidium iodide, and Hoechst 33,342

For the image flow cytometry assay, CF488A Annexin V and PI Apoptosis kit (Biotium, Fremont, CA, USA) and Hoechst 33,342 (ChemoMetec, Gydevang, Lillerød, Denmark) were used to detect cells in early apoptotic and late apoptotic stages. After staining, the cells were analyzed in NucleoCounter NC-3000 (ChemoMetec, Gydevang, Lillerød, Denmark) and NucleoView NC-3000 for automated image flow cytometry analysis.

### Western blotting

The cells were harvested 24 h after transient transfection, washed with phosphate-buffered saline, and subjected to lysis in radioimmunoprecipitation assay buffer (50 mM Tris–HCl pH 8.0, 150 mM NaCl, 1% NP40, 0.5% deoxycholate, 0.1% sodium dodecyl sulfate) at 4 °C for 30 min. Lysed cells were collected and centrifuged to remove cell debris. Protein assays were performed with the Bio-Rad Protein Assay kit based on the Bradford dye-binding procedure (Bio-Rad). Protein samples (40 μg) were separated by sodium dodecyl sulfate polyacrylamide gel electrophoresis in 10% or 12% resolving gel using a Mighty Small II Deluxe Mini Vertical Electrophoresis Unit (Hoefer, Inc., Holliston, MA, USA). Proteins were then electrotransferred to polyvinylidene difluoride membranes (PerkinElmer, Inc., Waltham, MA, USA) by Mighty Small Transfer Tank (Hoefer, Inc., Holliston, MA, USA). Subsequently, the membranes were blocked with a blocking buffer (50 mM Tris–HCl pH 7.6, 150 mM NaCl, 0.1% Tween 20, 5% nonfat dry milk, 0.05% sodium azide) for 1 h at room temperature and incubated with the following primary antibodies overnight at 4 °C: CCNA2 (1:1000; 18,202–1-AP, Proteintech Group, Inc., Rosemont, IL, USA), CCNB1(1:1000; 55,004–1-AP, Proteintech Group, Inc., Rosemont, IL, USA), CCND1 (1:1000; RM-9104-S, Thermo Fisher Scientific Inc., Waltham, MA, USA), CDK4 (1:1000; MS-299-P, Thermo Fisher Scientific Inc., Waltham, MA, USA), CDKN1B (1:1000; #3686, Cell Signaling Technology, Inc., Beverly, MA, USA), CDKN1A (1:1000; #2947, Cell Signaling Technology, Inc., Beverly, MA, USA), PARP (1:1000; sc-8007, Santa Cruz Biotechnology, Inc. Santa Cruz, CA, USA), CASP3 (1:1000; #9662, Cell Signaling Technology, Inc., Beverly, MA, USA), E2F1 (1:200, sc-251, Santa Cruz Biotechnology, Inc., Santa Cruz, CA, USA), PI3K p85 (1:1000; #4292, Cell Signaling Technology, Inc., MA, USA), AKT (1:1000; #4691, Cell Signaling Technology, Inc., MA, USA), Phospho-AKT (Thr308) (1:1000; #4056, Cell Signaling Technology, Inc., MA, USA), Phospho-AKT (Ser473) (1:1000; #4060, Cell Signaling Technology, Inc., MA, USA), GSK3β (1:1000; #9315, Cell Signaling Technology, Inc., MA, USA), Bcl-2 (1:1000; #4223, Cell Signaling Technology, Inc., MA, USA), BAX (1:1000; #5023, Cell Signaling Technology, Inc., MA, USA), Bad (1:1000; #9239, Cell Signaling Technology, Inc., MA, USA), Phospho-Bad(Ser136) (1:1000; #4366, Cell Signaling Technology, Inc., MA, USA), β-catenin (1:4000; C2206, Sigma-Aldrich, Inc., Beverly, MO, USA) and ACTB (1:2000, MAB1501, EMD Millipore, Billerica, MA, USA). The membranes were then incubated with anti-rabbit (sc-2004) or anti-mouse (sc-2005) immunoglobulin G horseradish peroxidase-conjugated secondary antibodies (1:10000, Santa Cruz Biotechnology, Inc., Santa Cruz, CA, USA) for 1 h at room temperature. After three washes with Tris-buffered saline with Tween-20 buffer (50 mM Tris–HCl pH 7.6, 150 mM NaCl, 0.1% Tween-20), immunoreactive bands were detected using a WesternBright ECL (Advansta, Menlo Park, CA, USA).

### Statistical analysis

The expression levels of Linc00659 in colon cancer from TCGA database were analyzed using Student *t* tests. The correlation of Linc00659 with the protein-coding genes in colon cancer was determined through Pearson coefficient analysis, with *r* and *p* values as indicated. Cumulative survival curves were estimated using the Kaplan–Meier method, and comparison between survival curves was performed using the log-rank test. The difference was considered significant when *p* < 0.05. The data on the expression levels of lncRNAs in paired colon tissues were analyzed using a paired *t* test. Cell proliferation, colony formation, and soft agar assay experiments were performed in triplicate. Histograms present the mean values, and the error bars indicate the standard deviation. These data were analyzed using Student *t* tests.

## Results

### Generation of lncRNA expression profiles in CRC

**Fig. 1:**
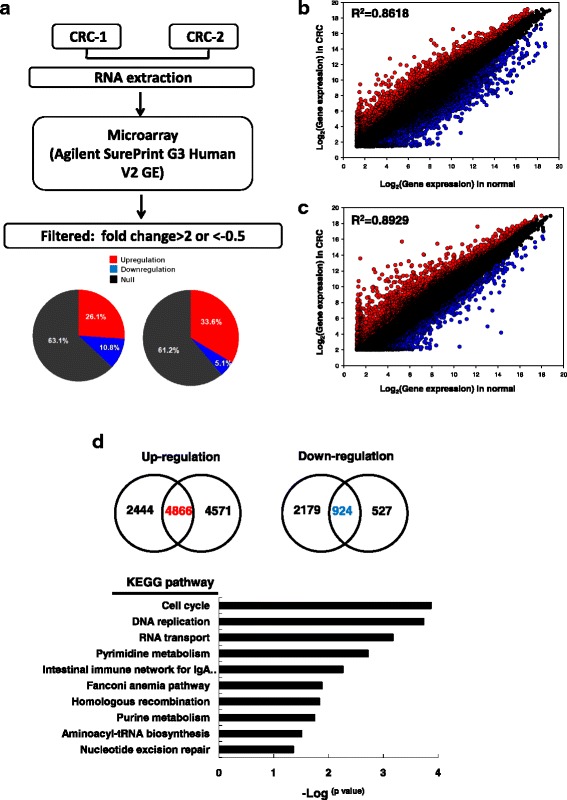
Transcriptome profiles of two CRC pairs were analyzed by a microarray approach. **a** Flowchart of the identification of differentially expressed genes through a microarray approach. Genes with differential expression in CRC compared with adjacent normal samples were filtered in terms of fold change ≥ 2 or fold change < 0.5. **b** and **c** Scatter plot of the distribution of all protein-coding genes; CRC tissues versus corresponding adjacent normal tissues from CRC#1 and CRC #2. **d** Venn diagrams of the number of upregulated and downregulated protein-coding gene candidates in the two N-T-paired CRC samples. Protein-coding genes with differential expression were subjected to pathway enrichment analysis. CRC, colorectal cancer

**Fig. 2:**
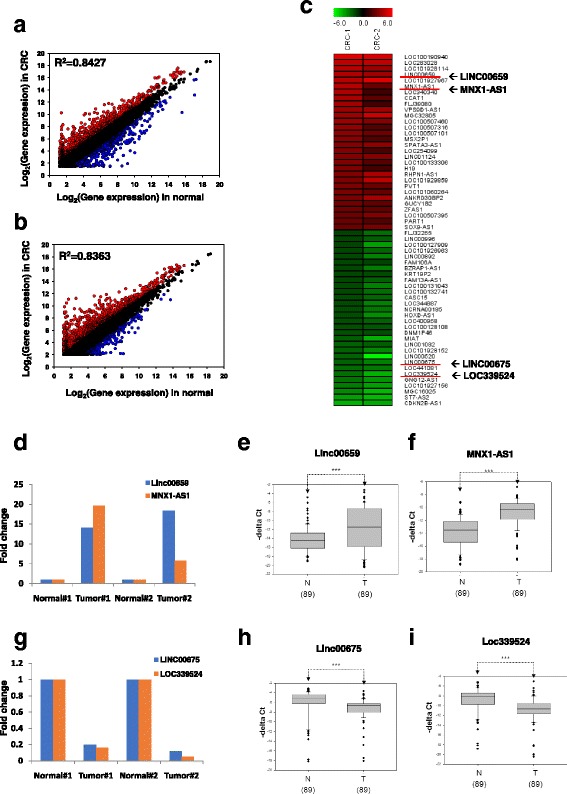
Identification of lncRNA candidates with differential expression in CRC from two transcriptome profiles. **a** and **b** Scatter plots of the lncRNA distribution; CRC tissues versus corresponding adjacent normal tissues from CRC#1 and CRC#2. **c** The 20 most aberrantly regulated lncRNAs candidates in CRC compared with adjacent normal samples were filtered in terms of fold change ≥ 4 or fold change < 0.25. **d**–**f** The expression levels of two upregulated lncRNA candidates, namely Linc00659 and MNX1-AS1, were determined to be differentially expressed in the CRC samples compared with the corresponding adjacent normal samples of two patients by using a microarray approach; this was validated using RT-qPCR in tissues of 89 patients. **g**–**i** The expression levels of two downregulated lncRNA candidates, namely Linc00675 and Loc339524, were determined to be differentially expressed in the CRC samples compared with the corresponding adjacent normal samples of two patients by using a microarray approach; this was validated using RT-qPCR in tissues of 89 patients. CRC, colorectal cancer; lncRNA, long noncoding RNAs; RT-qPCR, reverse transcription-quantitative polymerase chain reaction

We determined the gene expression profiles of the primary tumor and the corresponding adjacent normal tissues in two patients with CRC by using a microarray approach, in which protein-coding genes and lncRNAs were included (Agilent SurePrint G3 Human V2 GE; Fig. 1a). Most genes were consistently expressed between the CRC and the corresponding adjacent normal tissues in the two patients (*R*^2^ > 0.86; Fig. 1b and c). In total, 5790 protein-coding genes were detected as differentially expressed (fold change ≤ 2 or ≥ 0.5) between CRC and the corresponding adjacent normal mucosa, comprising 4866 upregulated genes and 924 downregulated genes, respectively (Fig. 1d, upper panels). We further examined the putative biological function of these differentially expressed genes by using pathway enrichment analysis. Our data revealed that these differentially expressed genes were significantly enriched in cell cycle, DNA replication, RNA transport, and pyrimidine metabolism signaling pathways (Fig. 1d, bottom panel). Abnormal lncRNAs in CRC were also identified by analyzing the same microarray data. Consistent with protein data, most lncRNAs were consistently expressed between the CRC and the corresponding adjacent normal tissues in the two patients with CRC (*R*^2^ > 0.83; Fig. 2a and b). Only a small subset of lncRNAs was differentially expressed between CRC and the corresponding adjacent normal tissues. A total of 201 lncRNAs were either upregulated (*n* = 165; fold change ≥ 4) or downregulated (*n* = 36; fold change ≤ 0.25) in the two CRC cases.

### LncRNA candidates were confirmed in colon cancer

The top 10 of these abnormal lncRNAs upregulated and downregulated are presented in Table 1. To validate the microarray analysis results, 4 of the top 10 differentially expressed lncRNAs were selected for validation in 89 matched CRC samples by using reverse transcription-quantitative PCR. Our results indicated that the expression levels of Linc00659 and MNX1-AS1 were significantly increased, whereas those of Loc339524 and Linc00657 were significantly decreased in colon cancer (Fig. 2d–i), which were consistent with our microarray results.

**Table 1:** Microarray analysis of the 20 most aberrantly regulated lncRNAs in two pairs of CRC

Probe name	lncRNA ID	Gene symbol	FC		Regulation	Chromosom
			CRC#1	CRC#2		
A_33_P3287399	ENST00000567788	LOC100190940	41.78	35.49	Up	chr12:130518100–130518041
A_21_P0001792	XR_249156	LOC101927967	20.28	39.41	Up	chr2:78245290–78245231
A_33_P3248231	XR_171099	LOC283028	22.19	22.93	Up	chr10:43248938–43248879
A_19_P00806771	ENST00000412500	LINC00659	14.13	18.42	Up	chr20:61405554–61405495
A_21_P0002735	ENST00000424786	LOC101928114	20.75	12.99	Up	chr3:34450773–34450832
A_32_P185317	ENST00000480284	MNX1-AS1	19.72	5.71	Up	chr7:156809059–156809118
A_33_P3531373	ENST00000602761	LOC340340	17.35	3.02	Up	chr7:106423149–106423208
A_21_P0005628	ENST00000500112	CCAT1	16.68	4.73	Up	chr8:128221174–128221115
A_33_P3347437	NR_033830	FLJ39080	14.33	3.11	Up	chr8:75670332–75670391
A_21_P0014466	XR_245037	LOC101928152	5.04	9.27	Down	chr2:87777086–87777027
A_32_P189781	ENST00000560267	LINC00520	5.09	54.58	Down	chr14:56247937–56247878
A_21_P0009298	ENST00000581851	LINC00675	5.11	8.11	Down	chr17:10698291–10698232
A_32_P49668	NR_073404	LOC441081	5.94	7.22	Down	chr5:68932239–68932298
A_32_P112592	ENST00000490006	LOC339524	6.22	18.77	Down	chr1:87634786–87634845
A_33_P3232930	ENST00000420587	GNG12-AS1	6.74	11.73	Down	chr1:68668599–68668658
A_21_P0001781	ENST00000435411	LOC101927156	7.71	13.70	Down	chr2:182263732–182263791
A_23_P79572	NR_026664	MGC16025	9.49	21.02	Down	chr2:240115646–240115587
A_33_P3365978	ENST00000434993	ST7-AS2	13.60	20.95	Down	chr7:116752410–116752351
A_19_P00322815	ENST00000585267	CDKN2B-AS1	18.31	16.36	Down	chr9:22118707–22118766

*CRC* colorectal cancer, *lncRNA* Long non-coding RNA, *ID* identification no, *FC* fold-change

### Linc00659 coexpressed with cycle-related genes

**Fig. 3:**
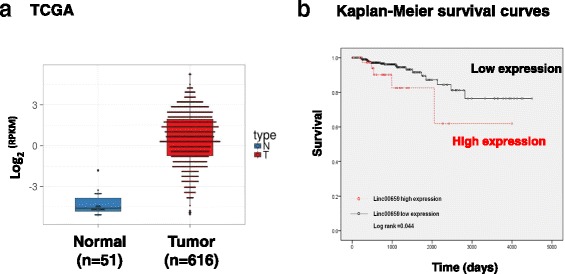
The clinical effects of Linc00659 expression were assessed by analyzing TCGA database. **a** The expression data of Linc00659 in 616 CRC tissues compared with 51 corresponding adjacent normal tissues were obtained from TCGA database. Boxplots of log2-transformed (RPKM) gene expression values for Linc00659 in CRC are shown. **b** Kaplan–Meier curves for overall survival time in patients with CRC, as determined according to Linc00659 expression. CRC, colorectal cancer; RPKM, reads per kilobase of transcript per million mapped reads; TCGA, The Cancer Genome Atlas

**Fig. 4:**
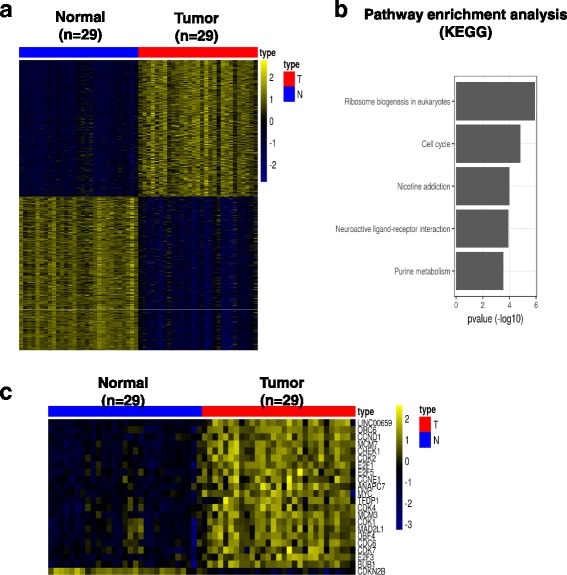
Identification of coexpression signatures associated with Linc00659 in CRC. **a** The correlation between protein-coding genes and Linc00659 was analyzed in 29 N-T-paired CRC samples by conducting Pearson correlation analysis. Expression levels of 1218 protein-coding genes were significantly correlated with Linc00659 with *r* ≥ 0.7 or ≤ − 0.7. **b** Coexpressed genes were subjected to pathway enrichment analysis, which revealed that these genes were enriched in the growth-related signaling pathway. **c** Heat map showing that the expression levels of cell cycle-related genes were well correlated with those of Linc00659 in 29 patients with CRC

The biological and clinical effects of Linc00659 expression were further examined by analyzing TCGA database. A total of 667 transcriptome data items were downloaded from TCGA database, comprising 51 normal and 616 CRC tissues. As shown in Fig. 3a, the expression levels of Linc00659 significantly increased in colon cancer compared with normal mucosa. Furthermore, high expression levels of Linc00659 were significantly correlated with a short survival curve of patients with CRC (Fig. 3b), implying that Linc00659 might play an oncogenic role in colon cancer progression. We further explored the biological role of Linc00659 in colon cancer tumorigenesis by identifying coexpression gene networks. A total of 1218 protein-coding genes were coexpressed with Linc00659 in colon cancer (*r* > 0.7 or <− 0.7; Fig. 4a). By determining these signaling pathways of the protein-coding genes coexpressed with Linc00659, we found that these highly interconnected genes were enriched in several cell proliferation-related signaling pathways (Fig. 4b). Notably, we also found that Linc00659 expression had a highly positive correlation with cell cycle-related gene expression in colon cancer, implying that Linc00659 might have an oncogenic role in colon cancer cell growth by accelerating cell cycle progression (Fig. 4c).

### Linc00659 was involved in colon cancer growth

**Fig. 5:**
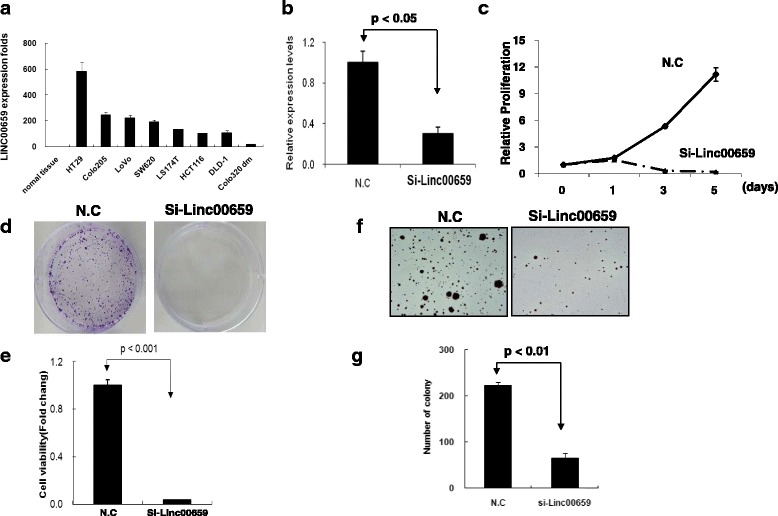
Knockdown of Linc00659 expression with siRNA suppressed HCT116 cell growth. **a** The expression levels of Linc00659 in eight colon cancer cell lines and adjacent normal tissues were showed. The expression levels of adjacent normal mucosal tissue were obtained from Fig. 2e. **b** The expression levels of Linc00659 were examined after transfection of HCT116 cells with siRNA. **c** and **d** After transient transfection of HCT116 cells with si-Linc00659, cell proliferation and colony formation assays were performed: **e** graph illustrating quantified values from the colon formation assays. **f** The anchorage-independent growth of HCT116 with si-Linc00659 transfection was examined by a soft agar formation assay: **g** graph illustrating quantified values from the assay. Data are reported as the number of colonies relative to the control (mean ± standard deviation). siRNA, small interfering RNA

**Fig. 6:**
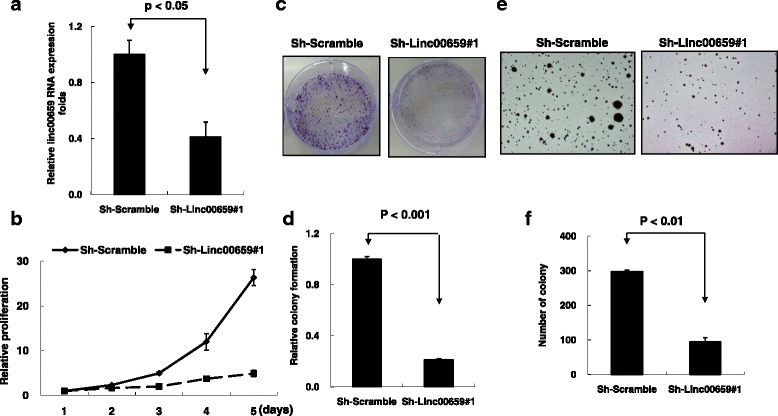
Stable knockdown of Linc00659 expression with shLinc00659#1 suppressed HCT116 cell growth. **a** The expression levels of Linc00659 were examined in HCT116 cells with stable Linc00659 knockdown. **b** and **c** The cell proliferation and colony formation assays were performed: **d** graph illustrating quantified values. **e** The effects of Linc00659 knockdown on anchorage-independent growth of HCT116 were examined by a soft agar formation assay: **f** graph illustrating quantified values. Data are reported as the number of colonies relative to the control (means ± standard deviation). shRNA, short hairpin RNA

We examined the expression levels of Linc00659 in eight colon cancer cell lines, namely colo320dm, colo205, DLD1, Lovo, SW620, HCT116, LS174T, and HT29, by using real-time PCR. Compared with adjacent normal mucosa, the expression levels of Linc00659 were clearly overexpressed in eight colon cancer cell lines by more than 20-fold (Fig. 5a). Based on this result, we selected a loss-of-function approach to study the biological function of Linc00659 in colon cancer cells. As demonstrated in Fig. 5b, Linc00659 expression significantly decreased by 70% in HCT116 cells after siRNA transfection.. Knockdown of Linc00659 expression significantly suppressed cancer cell proliferation and colony formation in HCT116 (Fig. 5c–e). Using a soft agar assay, we indicated that Linc00659 knockdown could significantly suppress anchorage-independent growth in HCT116 cells (Fig. 5f and g). We also observed that the cell proliferation of Lovo, LS174T, DLD-1 and SW620 could be suppressed after Linc00659 knockdown for 4 days (Additional file 1: Figure S1). We further established stable knockdown of Linc00659 expression through sh-Linc00659 transfection and puromycin selection for 2 weeks. As illustrated in Fig. 6a and Additional file 2: Figure S2A, the expression levels of Linc00659 were significantly decreased by 60% in sh-Linc00659 compared with the control. The stable knockdown of Linc00659 expression exhibited similar effects on cell growth in accordance with si-Linc00659 treatment. The cell proliferation, colony formation, and anchorage-independent growth abilities were significantly decreased in HCT116 cells with stable Linc00659 knockdown (Fig. 6b–f and Additional file 2: Figure S2B-F).

### Knockdown of Linc00659 suppressed cell growth by impairing cell cycle progression and inducing cell apoptosis

**Fig. 7:**
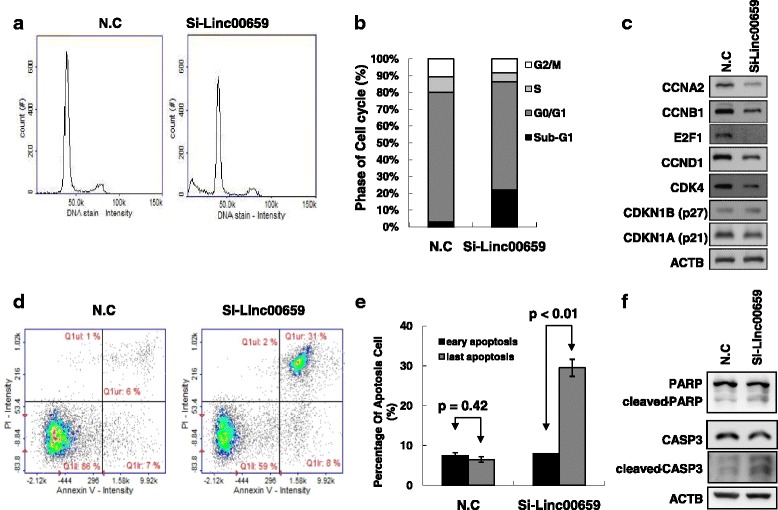
Linc00659 knockdown suppressed colon cancer cell proliferation by impairing cell cycle progression and inducing apoptosis. **a** and **b** Distribution of cells in three cell cycle phases was examined by an image flow cytometry assay, and the graph shows quantification for each phase. **c** The expression levels of cell cycle-relative protein were examined by a Western blot assay. **d** For measurement of apoptotic cells, cells were stained with both propidium iodide and Annexin V and analyzed by an image flow assay: **e** graph illustrating the quantification of apoptotic cells (**e**). **f** The expression levels of apoptosis-relative protein were examined by a Western blot assay

Our results revealed that Linc00659 could regulate colon cancer growth. Coexpression data indicated that Linc00659 had a high correlation with cell cycle-related genes; therefore, we explored the mechanism underlying the regulation of cell growth by examining the cell cycle distribution and apoptosis after Linc00659 knockdown. Image flow assay results indicated that the cell cycle distribution was disrupted after Linc00659 knockdown. Transient knockdown of Linc00659 cells also indicated that the G_1_ phase decreased in HCT116 cells (Fig. 7a and b). Furthermore, an examination of the cell cycle-related protein expression levels revealed that the expression levels of cyclin A2, cyclin B1, cyclin D1, CDK4, and E2F1 decreased in HCT116 cells with Linc00659 knockdown (Fig. 7c). Notably, the sub-G_1_ population cells were obviously increased in Linc00659 cells subjected to transient knockdown, including HCT116 (Fig. 7a and b). Apoptosis assay results indicated that the apoptotic cells significantly increased and that PARP and caspase-3 cleavage was induced in HCT116 cells with si-linc00659 transfection (Fig. 7d–f). These data indicate that Linc00659 knockdown could impair cell cycle progression and induce cell apoptosis.

### Linc00659 knockdown confers drug sensitivity to colon cancer cells

**Fig. 8:**
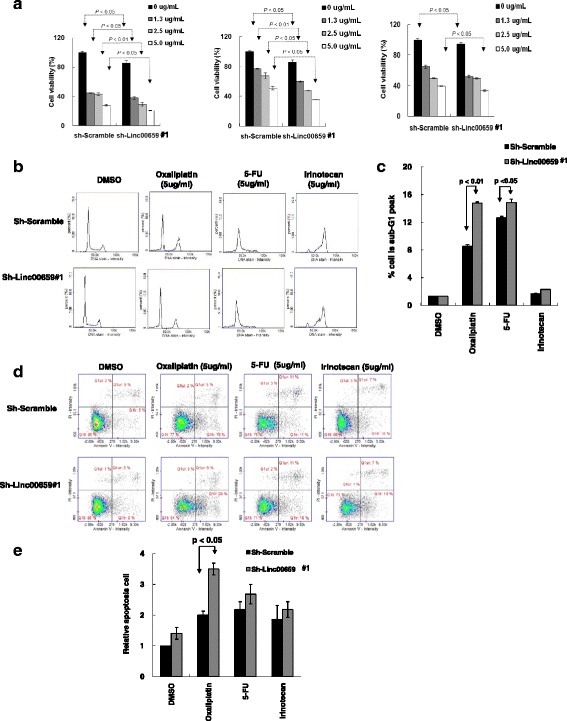
Linc00659 knockdown conferred colon cancer cell drug sensitivity. **a** HCT116 cells with shLinc00659#1 knockdown were exposed to various concentration (0, 1.3, 2.5 and 5.0 μg/ml) of oxaliplatin(left panel), 5-FU(middle panel), and irinotecan(right panel) for 48 h, respectively. Cell viability was then examined using CellTiter-Glo One Solution cell proliferation assay. **b** and **c** After exposure to 5 μg/mL oxaliplatin, 5-FU, and irinotecan for 48 h, the cell cycle distribution was examined by an image flow cytometry assay, and the percentage of sub-G1 was quantified in HCT116 cells with shLinc00659#1 knockdown and in control cells. **d** and **e** Cells were stained with both propidium iodide and Annexin V; apoptotic cells were analyzed by an image flow assay, and the percentage of apoptotic cells was quantified. 5-FU, 5-fluorouracil

To explore whether Linc00659 expression is involved in the regulation of drug responsiveness, we assessed the effects of Linc00659 knockdown on cell apoptosis in HCT116 with drug treatment, including oxaliplatin, 5-fluorouracil (5-FU), and irinotecan treatment. Linc00659 was stably knocked down and the respective control cells were exposed to various concentration of oxaliplatin, 5-FU, or irinotecan for 48 h, and cell viability was examined. As presented in Fig. 8a, Linc00659 knockdown could obviously accelerate the reduction of cell viability in HCT116 following oxaliplatin or 5-FU, and slightly reduced cell viability with irinotecan treatment. An examination of the cell cycle progression revealed that Linc00659 knockdown resulted in a significant increase of the sub-G_1_ phase of the cell cycle distribution after oxaliplatin and 5-FU treatment (Fig. 8b and c). In addition, Linc00659 knockdown increased apoptotic cells after oxaliplatin treatment compared to control group (Fig. 8d and e). Overall, the expression levels of Linc00659 conferred drug sensitivity in colon cancer by inducing apoptosis.

### Effects of Linc00659 knockdown on PI3K-AKT-GSK3β signaling

**Fig. 9:**
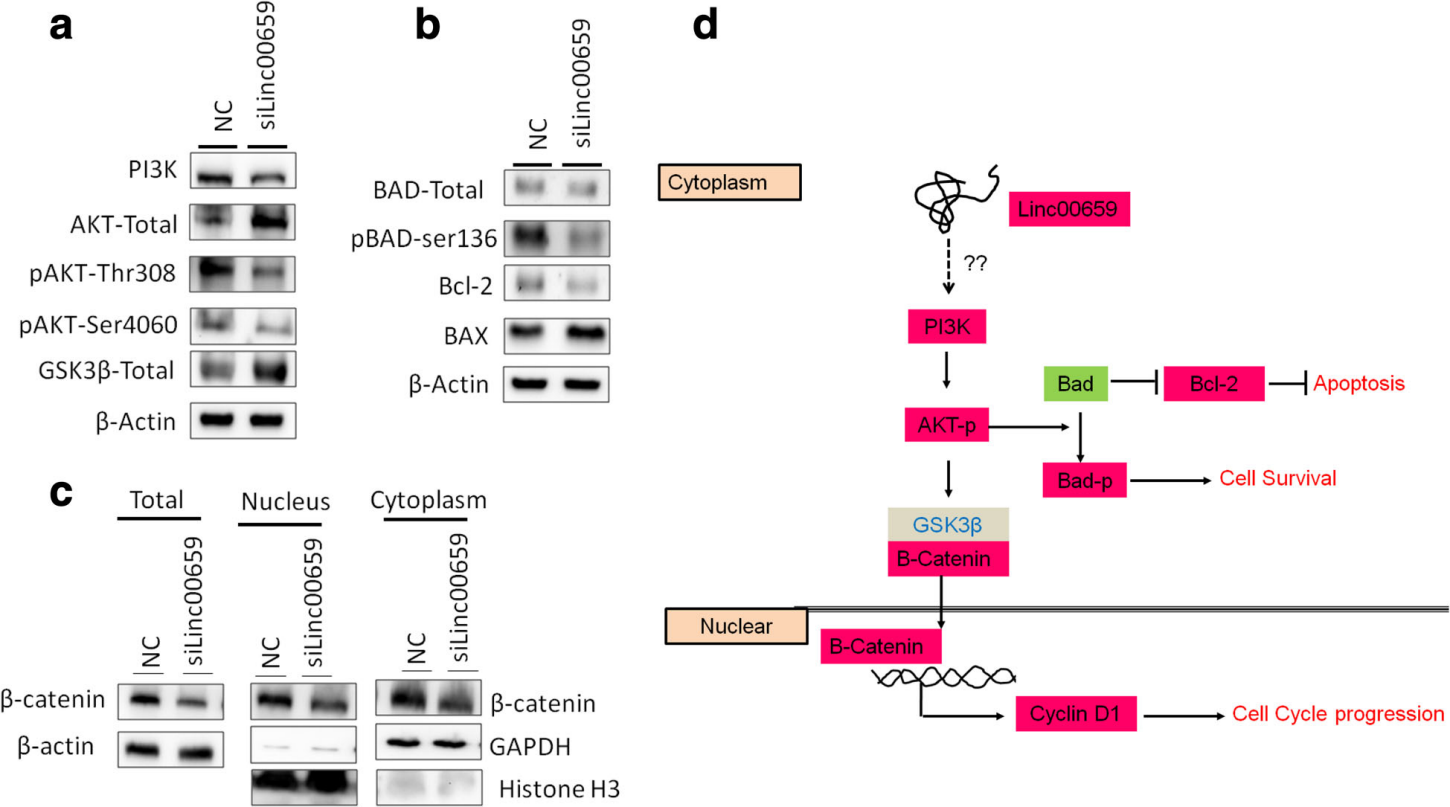
Linc00659 knockdown mediated PI3K-Akt signaling regulation leading to cell growth inhibition and cell death in CRC. **a** The expression levels of PI3K, Akt, phosphorylation of Akt and GSK3β were examined in HCT116 cells with Linc00659 knockdown. **b** The downstream effectors of PI3K-Akt pathway were examined by western blotting in HCT116 cells with Linc00659 knockdown. β-Actin was used for an input control. **c** the expression levels ofβ-catenin, cytoplasmic β-catenin and nuclear β-catenin were analyzed in HCT116 cells after Linc00659 knockdown. GAPDH was used as a cytoplasmic marker and Histone H3 was used as a nucleus marker. **d** Model for determining the role of Linc00659 in modulating the growth of colon cancer cells

Studies have indicated that AKT activation plays a crucial role in the cell-cycle progression and drug resistance of colon cancer [22–24]. Therefore, we sought to determine whether the P13K-AKT signaling pathway is involved in suppressing growth in HCT116 with Linc00659 knockdown. As shown in Fig. 9a, the protein levels of PI3K and p-AKT were decreased in HCT116 after Linc00659 knockdown. In addition, studies have indicated that AKT signaling acts has a crucial role in colon cancer cell survival by modulating BCl2 and Bad [25, 26]. Therefore, we examined these apoptosis-related proteins, revealing that the levels of anti-apoptosis proteins BCl2 and p-Bad both decreased after Linc00659 knockdown (Fig. 9b); we also observed that GSK3β expression was increased in Linc00659 knockdown compared with the control group. We further examined the expression levels of B-catenin, revealing that total protein and nuclear-B-catenin were decreased in HCT116 with Linc00659 knockdown (Fig. 9c). Taken together, our results demonstrate a putative mechanism of Linc00659 knockdown that impairs cell cycles and induces apoptosis; this may be a result of suppressing the PI3K-AKT-GSK3B axis in colon cancer.

## Discussion

Increasing evidence reveals that numerous lncRNAs play a critical role in the pathogenesis and progression of CRC. In the present study, transcriptome profiles were analyzed to identify differentially expressed lncRNA in colon cancer by using a microarray approach, which revealed that the expression levels of 201 lncRNAs were significantly different between colon cancer and the corresponding adjacent normal tissues. As expected, our microarray profiles identified several lncRNAs, which were aberrantly expressed in human cancer, such as LOC100190940, LOC100506178, H19, PVT1, VPS9D1-AS1, Linc00520, Linc00675, GNG12-AS1 CDKN2B-AS1, and CCAT1 [19, 27–36].

Chen et al. systematically examined global lncRNA dysregulation by analyzing RNA-seq data of colon cancer [36]. They identified that LOC100190940 was upregulated in primary tumor tissues compared with adjacent normal tissues, and that high LOC100506178 expression levels were correlated with poor survival in patients with colon cancer. In addition, they reported that Linc00659 was highly expressed in primary tumor tissues with a higher H3K4me3 signal in its promoter region, compared with normal tissues. This result implies that the expression levels of Linc00659 might be regulated by the epigenetic mechanism in CRC [36]. A previous study indicated that c-Myc could promote CCAT1 transcription by activating its promoter activity, and that ectopic CCAT1 expression could accelerate growth and invasion in colon cancer [19]. Furthermore, high CCAT1 expression levels were significantly associated with poor prognosis and survival in patients with colon cancer [19, 29]. In this study, we identified hundreds of novel abnormal lncRNA genes to provide valuable information for lncRNA research in colon cancer.

The mechanisms through which lncRNAs participate in carcinogenesis and CRC progression are various and complex. To date, the mechanism underlying lncRNA function remains largely unknown, and the molecules with which they interact or how their expression influences signaling is unclear. Although studies have attempted to predict the putative mechanism of dysfunctional lncRNAs by using the bioinformatics approach, the lack of conserved sequences and the absence of functional motifs have made it difficult to determine the function of lncRNAs by analyzing their sequence or secondary structures. Using transcriptome data to determine which types of protein-coding genes are coexpressed with lncRNAs may facilitate exploring lncRNA function [37]. In this study, we used TCGA database to determine coexpressed protein-coding genes and demonstrate the biological function of Linc00659 involved in modulating cancer cell growth. Using an experimental approach, we further confirmed that Linc00659 knockdown could significantly suppress colon cancer growth by impairing cell cycle progression and inducing cell apoptosis. Moreover, our data reveal that Linc00659 conferred drug sensitivity to colon cancer cells. Studies have increasingly indicated that some lncRNAs were associated with drug resistance in human cancer [38–40]. Lee et al. identified differentially expressed long intergenic noncoding RNAs (lincRNAs) in 5-FU-resistant colon cancer cells [39]. A total of 42 lincRNA candidates were identified with differential expression in 5-FU-resistant cells. Among them, colon cancer cells with snaR loss could decrease in vitro sensitivity to 5-FU [39]. In addition, 46 lncRNAs were identified with differential expression in vincristine-resistant colon cancer cells, compared with control cells, using next-generation sequencing profiling [40]. The expression levels of Linc00152 conferred colon cancer cell resistance to oxaliplatin by modulating the miR-193a-3p/ERBB4/AKT signaling axis [41]. In our study, we demonstrated that Linc00659 knockdown could accelerate cell apoptosis in colon cancer cells following oxaliplatin treatment. An analysis of the Gene Expression Omnibus database showed that the expression levels of Linc00659 were increased in drug-resistant breast and colon cancer cells [42, 43]. According to our present study, we determined a putative mechanism by which Linc00659 may play a novel oncogenic role in cell growth and drug resistance by modulating PI3K-AKT signaling.

Unlike protein-coding genes, whose transcripts are mainly exported to the cytoplasm for translation, lncRNAs are distributed in either the cytoplasm or the nucleus, depending on their function. Therefore, further examination of subcellular localization of lncRNA would facilitate determining the putative mechanism. We analyzed the subcellular localization of Linc00659, revealing that Linc00659 expression occurred both in the nucleus and cytoplasm in most colon cancer cells (Additional file 3: Figure S3). According to its location, Linc00659 might regulate colon cancer cell growth by binding with microRNA sponge, regulate transcription factors, or modulate epigenetic modification to prevent tumor suppressor gene expression. Notably, we observed that Linc00659 expression predominantly enriches in the nucleus compared with the cytoplasm in HT29 cells (Additional file 3: Figure S3). Studies have reported that the siRNA approach was less efficient for knockdown nuclear-lncRNA expression [44]. In the present study, we were unable to explore the role of Linc00659 in HT29 cells using the same siRNA oligo. According to the varying localization of Linc00659, we suggest that Linc00659 might play an oncogenic role through the difference mechanism in HT29 cells. However, the detailed mechanism of Linc00659 remains unclear.

## Conclusion

In this study, we identified lncRNAs with differential expression in colon cancer. We first reported that the expression levels of Linc00659 were significantly increased in colon cancer and that Linc00659 loss suppressed colon cancer cell growth by impairing cell cycle progression. Our finding also establishes Linc00659 as a prognostic biomarker for indicating CRC survival and to be used as adjuvant therapy for chemotherapy.

## Additional files

Additional file 1: **Figure S1.** Knockdown of Linc00659 expression with siRNA suppressed growth of colon cancer cells (A)-(D) The expression levels of Linc00659 were examined after transfection of Lovo, LS174T, DLD-1 and SW620 with siRNA. (E)-(H) After transient transfection of Lovo, LS174T, DLD-1 and SW620 cells with si-Linc00659, cell proliferation was examined; the graph shows quantified values. (DOCX 124 kb)

Additional file 2: **Figure S2.** Stable knockdown of Linc00659 expression with shLinc00659#2 suppressed HCT116 cell growth. (A) The expression levels of Linc00659 were examined in HCT116 cells with stable shLinc00659#2 knockdown. (B) and (C) The cell proliferation and colony formation assays were performed: (D) graph illustrating quantified values. (E) The effects of Linc00659 knockdown on anchorage-independent growth of HCT116 were examined by a soft agar formation assay: (F) graph illustrating quantified values. Data are reported as the number of colonies relative to the control (means ± standard deviation). (DOCX 205 kb)

Additional file 3: **Figure S3.** Examination of the subcellular fractionation localization of Linc00659 in CRC cell lines. After nuclear and cytosolic separation, total RNA from Lovo, HCT116, HT29, SW620 and DLD-1 cells underwent RT and real-time PCR. GAPDH was used as a cytosol marker (A) and U6 was used as a nucleus marker (B). (C) RNA expression levels of Linc00659 candidates in the nucleus and cytoplasm were measured by real-time PCR, respectively. CRC, colorectal cancer; GAPDH, glyceraldehyde 3-phosphate dehydrogenase; PCR, polymerase chain reaction. (DOCX 109 kb)
