# Supplementary material for: Linc00659, a long noncoding RNA, acts as novel oncogene in regulating cancer cell growth in colorectal cancer
Source: Mol Cancer. 2018 Mar 10;17:72. doi: 10.1186/s12943-018-0821-1 (PMC5845323; doi:10.1186/s12943-018-0821-1)
Supplement: Supplementary file 1 [file 12943_2018_821_MOESM1_ESM.docx]

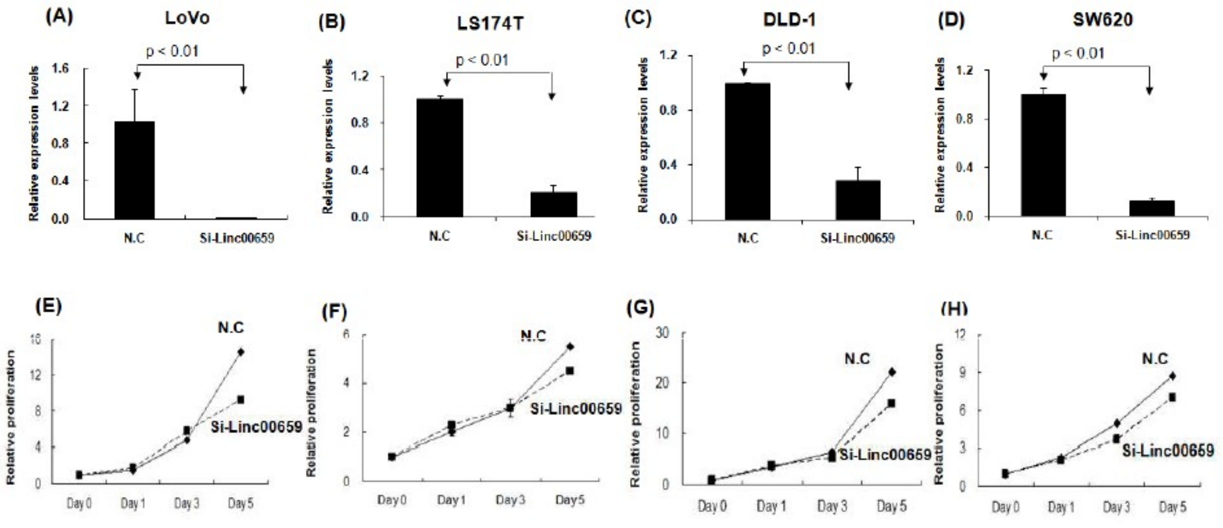

Supplementary Figure 1
